# The Catalytic Mechanism of Key Enzymes Involved in the Synthesis of Ergothioneine in *Lentinula edodes*

**DOI:** 10.3390/molecules29246005

**Published:** 2024-12-20

**Authors:** Zheng Li, Jianjun Ding, Wen Huang, Yinbing Bian, Xi Feng, Ying Liu

**Affiliations:** 1 College of Food Science & Technology, Huazhong Agricultural University, Wuhan 430070, China; lizheng123@webmail.hzau.edu.cn (Z.L.); huangwen@mail.hzau.edu.cn (W.H.); 2 School of Food Science and Technology, Jiangnan University, Wuxi 214122, China; 7230111018@stu.jiangnan.edu.cn; 3 Institute of Applied Mycology, Huazhong Agricultural University, Wuhan 430070, China; bianyinbing@mail.hzau.edu.cn; 4 Department of Nutrition, Food Science and Packaging, San Jose State University, San Jose, CA 95192, USA

**Keywords:** *Lentinula* *edodes*, ergothioneine, C-S lyase, biocatalysis

## Abstract

C-S lyase is a crucial enzyme responsible for the formation of sulfur-containing flavor compounds in *Lentinula edodes*. We investigated the involvement of C-S lyase in the synthesis of ergothioneine (EGT) in *L. edodes*, a high-producing edible mushroom. Through experimental and computational approaches, we identified *Lecsl*2, a C-S lyase, as a key enzyme involved in EGT synthesis in *L. edodes*. We characterized the enzymatic catalytic mechanism of Egt1 and Egt2, the two enzymes primarily catalyzing EGT synthesis in fungi. The results showed that Egt1 interacted with His, SAM, and Cys to form the intermediate product Her-sul, while Egt2, a PLP-dependent enzyme, cleaved the C-S bond on Her-sul to produce EGT. However, our findings suggested that Egt2 in *L. edodes* might not form a covalent bond with PLP, unlike the previously reported catalytic mechanism of Egt2 involving covalent catalysis. The study provided new insights into the synthesis pathway of EGT in *L. edodes* and highlighted the need for further investigation into the catalytic mechanism of Egt2 in this species.

## 1. Introduction

**Figure 1:**
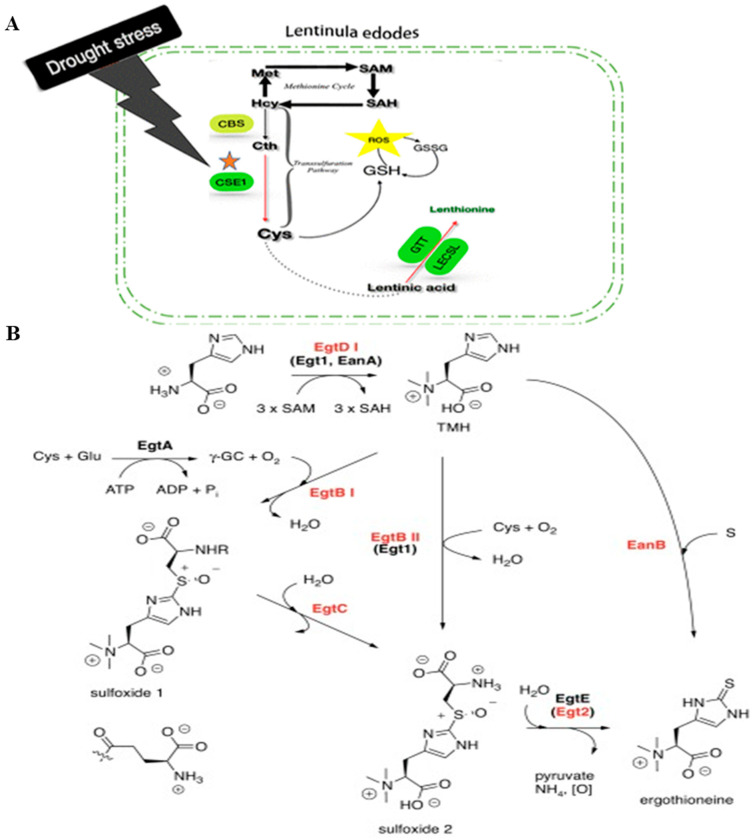
(**A**): Synthesis path of lenthionine [3]; (**B**): EGT synthesis pathways of fungi and bacteria [14].

*Lentinula edodes*, one of the highest-producing edible mushrooms in the world, stands apart from other edible mushrooms due to its unique properties. When dried, it emits a distinct odor, attributed to sulfur-containing compounds like lenthionine, which impart its characteristic scent [1,2]. It is widely believed that Gamma glutamyl transpeptidase (GGT) and L-cysteine sulfoxide lyase (C-S lyase) play pivotal roles in the formation of these sulfur-containing odor components in *L. edode*s as shown in Figure 1 [3,4,5]. Ergothioneine (EGT), an odorless and colorless sulfur compound, possesses potent hydroxyl radical scavenging, DPPH radical scavenging, and ABTS radical scavenging capabilities. Its versatility has led to its application in various fields, including food and cosmetics [6,7]. In fungi, the synthesis of EGT is primarily catalyzed by two enzymes: Egt1 and Egt2 (Figure 1). These enzymes utilize histidine, S-adenosylmethionine (SAM), and cysteine as substrates. Specifically, Egt1 is a SAM-dependent methyltransferase, which transfers the three methyl groups on SAM to histidine to form hercynine, which then binds with cysteine to form the Cys-HER complex (Hercynylcysteine superoxide, Her-sul). On the other hand, Egt2, a pyridoxal phosphate (PLP)-dependent C-S lyase, cleaves the C-S bond on Her-sul to produce EGT [8,9,10]. Research indicated that Egt2 exhibits cysteine sulfoxide lyase activity. The key enzyme C-S lyase, involved in the formation of sulfur-containing odor components in *L. edodes*, bears similarities in activity to Egt2 [11,12,13]. However, whether C-S lyase plays a role in the synthesis of EGT in *L. edodes* remains an area of further investigation. In this study, we primarily investigated whether the key enzyme C-S lyase, which synthesizes sulfur-containing characteristic odor components in *L. edodes*, is also involved in the synthesis of ergothioneine. Based on this, we provided a brief analysis of the enzymatic catalytic mechanism of the key enzymes involved in ergothioneine synthesis in *L. edodes*. By deciphering the intricate link between the sulfur-containing aroma compounds in *L. edodes* and EGT synthesis, this research aimed to deepen the understanding of the biosynthetic mechanisms underlying sulfur-containing secondary metabolites in *L. edodes*. Through rigorous validation of the functions of the relevant genes, this study laid a robust scientific foundation for advancing the genetic breeding of *L. edodes*.

## 2. Results and Discussion

### 2.1. Synthesis Pathway of EGT in L. edodes

**Figure 2:**
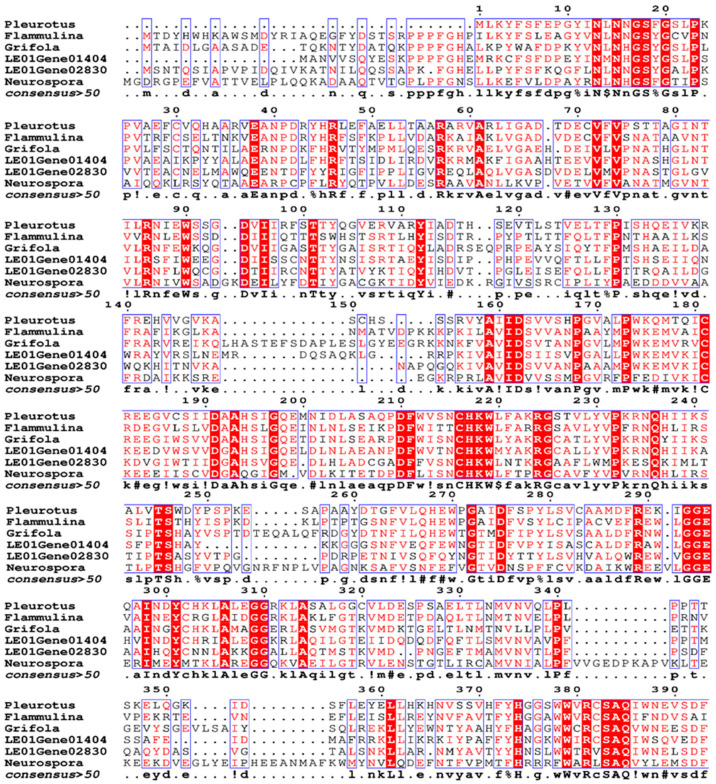
Sequence alignment by ESPript [21] of *Lecsl*1 (LE01Gene01404) and *Lecsl*2 (LE01Gene02830) in *L. edodes* with Egt2 in fungi.

The Egt1 gene (LE01Gene 12188) was first identified by aligning the reference genome of *L. edodes* W1-26 [7,15]. Upon sequence alignment, it was revealed that the *Lecsl*1 (LE01Gene01404) and *Lecsl*2 (LE01Gene02830) genes in the reference genome of *L. edodes* W1-26 belonging to the C-S lyase family bear a striking resemblance to the Egt2 amino acid sequences found in fungi [16,17] (Figure 2). In the following, we referred to the fungal EGT synthesis pathway [18] and formulated a solution consisting of the following substrate and cofactors: 0.1 M His, 0.1 M SAM, 0.01 M Cys, 0.1 M FeSO_4_·7H_2_O, and 0.01 M PLP. Tris-HCl (pH 8.0) served as the buffer for this mixture. Two proteins (Egt1 and *Lecsl*1) were incorporated into the reaction group, while a non-protein system functioned as the blank control. After preparing, the reaction solution was taken in a water bath maintained at 30 °C for two hours. However, liquid chromatography-mass spectrometry (LC-MS) facilitated the detection of EGT. Although this experiment did not produce EGT, the intermediate product Her-sul was discernible in the reaction group, whereas it remained undetected in the blank group (*m*/*z* = 333.12756). Through a C-S lyase activity assessment, it transpired that the recombinant protein *Lecsl*1 lacked C-S lyase activity. This absence could be attributed to several factors: (1) an unsuitable expression vector; or (2) proteins expressed in the prokaryotic system (*Escherichia coli*) that have not undergone folding and post-translational modifications, whereas proteins derived from eukaryotic organisms necessitate such modifications for proper functionality [19].

Not only did we fail to detect EGT, but we also failed to identify Hercynine in this experiment. This could potentially be explained by the immediate binding of Hercynine with Cys to form Her-sul under the catalysis of Egt1 upon its formation, or it might be due to the concurrent binding of both the methyl group and Cys to His, given that Egt1 possesses domains for both methyltransferase and cysteine sulfoxide synthase [6,20].

**Figure 3:**
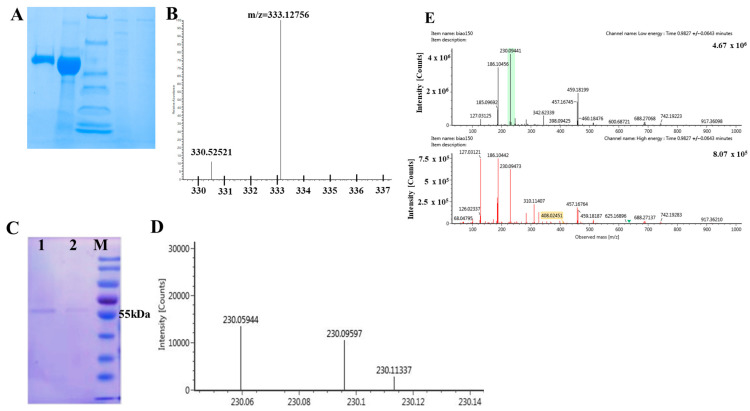
(**A**): Protein SDS-PAGE gel plot, sample wells from left to right are as follows: purified *Lecsl*1 protein, concentrated *Lecsl*1 protein, protein marker, band size from top to bottom: 116, 66.2, 45, 35, 25, 18.4, 14.4 KDa, concentrated Egt1 protein, purified Egt1 protein; (**B**): Mass spectrometry of Her-sul; (**C**): Recombinant *Lecsl*2 protein SDS-PAGE, 1-2: recombinant protein, M: protein marker; (**D**): Mass spectrum of EGT in the reaction group; (**E**): Mass spectrum of EGT standard, the green label indicates the m/z of the EGT standard.

Our previous studies have demonstrated that the *Lecsl*2 protein, when expressed in *Escherichia coli*, possessed C-S lyase activity, which was activated by pyridoxal phosphate (PLP) [22,23]. In light of this, we expressed the *Lecsl*2 protein in *Escherichia coli* to ascertain its role in the production of EGT. In addition, we also optimized the reaction system. We referred to the synthesis pathway of EGT in *Neurospora crassa* [10] and induced the expression of *Lecsl*2 protein by adding PLP to the culture medium, achieving a final concentration of 0.1mM. Drawing from the existing literature on EGT synthesis, we divided the in vitro reaction into two distinct steps. We first introduced the Egt1 protein and incubated the mixture at 32 °C for two hours. Following this, we added the recombinant *Lecsl*2 protein along with the antioxidant dithiothreitol (DTT). After a further hour of incubation at 30 °C, we terminated the reaction by adding methanol. A non-protein system was set as the control, and LC-MS was also employed to ascertain the presence of EGT in both the reaction and control groups. The result is illustrated in Figure 3. We detected the production of EGT in the reaction group by the LC-MS analysis, albeit in relatively low quantities, based on molecular weight information derived from the mass spectrum and corroborated with standard references and the existing literature. Therefore, our experimental findings suggest that the key enzyme C-S lyase, implicated in the formation of sulfur-containing odor components, also played a role in the synthesis of EGT in *L. edodes*.

### 2.2. The Enzymatic Catalytic Mechanism of Egt1 and Egt2

**Figure 4:**
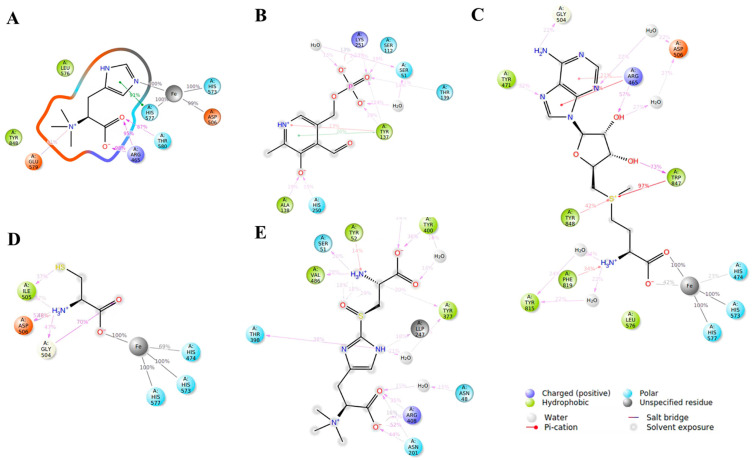
Schematic diagram of key enzymes and substrate binding in the synthesis pathway of EGT in *L. edodes*. (**A**): Egt1-Fe^2+^-His; (**B**): Egt2-PLP; (**C**): Egt1-Fe^2+^-SAM; (**D**): Egt1-Fe^2+^-Cys; (**E**): Egt2-PLP-Her-sul.

**Figure 5:**
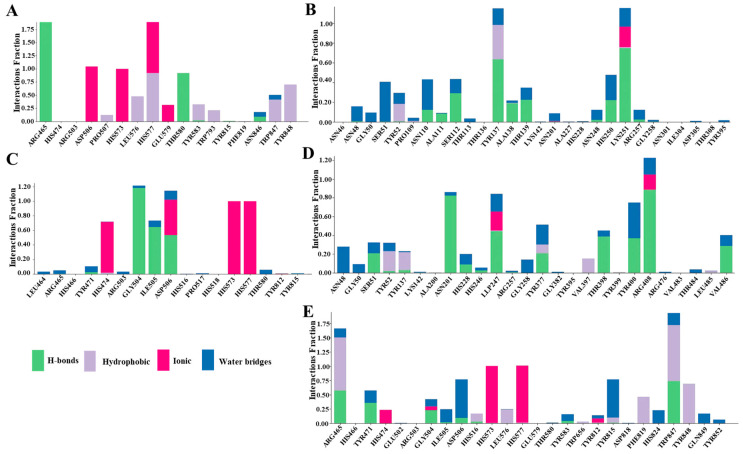
The key enzymes in the synthesis pathway of EGT in *L. edodes* bind to the amino acid sites and modes of action of substrates. (**A**): Egt1-Fe^2+^-His; (**B**): Egt2-PLP; (**C**): Egt1-Fe^2+^-Cys; (**D**): Egt2-PLP-Her-sul; (**E**): Egt1-Fe^2+^-SAM.

After proving the functions of Egt1 and Egt2 in *L. edodes* through enzyme activity experiments, we explored their mechanisms of action with substrates through molecular simulation. Figure 4 and Figure 5 illustrate the interactions between Egt1 and Egt2 with their substrates in *L. edodes*, respectively. Since Egt1 utilized Fe^2+^ as a cofactor during the reaction, we initially constructed a model of the Egt1-Fe^2+^ complex, and we found that Fe^2+^ primarily formed a complex with Egt1 by binding to His-573 and His-577. Subsequently, we performed molecular docking of this complex model with the substrates His, SAM, and Cys, respectively. Based on the results of molecular docking, we identified the primary amino acid binding sites and conducted an analysis of the binding modes between amino acid residues and the substrate. Arg-465, His-577, and Thr-580 of Egt1 formed hydrogen bonds with histidine, while Asp-506 bonded to Fe^2+^ during this process. However, when the substrate was SAM, Asp-506 of Egt1 did not bind to Fe^2+^. Arg-465 of Egt1 also interacted with SAM, not only through hydrogen bonds but possibly through hydrophobic interactions as well. Compared to Arg-465, Trp-847 appeared to bind more tightly to SAM, exhibiting higher interaction scores for both hydrogen bonds and hydrophobic interactions than Arg-465.The binding site of Trp-847 was located near the methyl group of SAM, and Egt1 possessed a conserved domain characteristic of methyltransferases, thus it might be the site where Egt1 exerted its methyltransferase activity. The binding sites for Cys and Egt1 were Gly-504 and Asp-506. Gly-504 formed a hydrogen bond with Cys, while Asp-506 exhibited multiple binding modes with Cys. 

Through molecular docking, we pinpointed the crucial amino acid residue of Egt1 from *L. edodes*: Arg-465, Gly-504, Asp-506, His-573, His-577, and Trp-847. Based on the previously reported biosynthetic pathway of fungal EGT, Egt1 initially transferred the methyl group from SAM to His to form Hercynine, which subsequently combined with Cys to produce HER-sul. In this study, Hercynine was not detected by LC-MS. Our molecular docking results revealed that when Egt1 interacted with His, Asp-506 bonded to Fe^2+^. Notably, ASP-506 was also the site on Egt1 that bonded to Cys. When Asp-506 bonded to Cys, it did not bind to Fe^2+^. This suggested that Egt1 acted on His first, and then interacted with Cys. The failure to detect Hercynine in this study might be attributed to the rapid formation of Her-sul from Hercynine catalyzed by Egt1.

After delving into the interaction mechanism between Egt1 and its substrate, we proceeded to investigate the binding mechanism of Egt2 with its substrate. Given that Egt2 is a PLP-dependent enzyme, our initial step was to perform the molecular docking of Egt2 with PLP. The docking results revealed multiple potential binding sites on Egt2 for PLP interaction. Previous molecular docking studies of C-S lyase from *L. edodes* (referred to as Egt2 in this context) with PLP had identified Ser-112, Asp-225, His-228, Asn-248, His-250, and Lys-251 as the binding sites on the protein for PLP. Upon re-docking using a different algorithm, we found that Asn-248, His-250, and Lys-251 on Egt2 continued to interact with PLP, with Lys-251 exhibiting a strong binding affinity to PLP. This finding suggested that the region on Egt2 that binds to PLP was centered around Lys-251. However, upon docking the Egt2-PLP complex with the substrate Her-sul, we found that Egt2-PLP failed to bind with Her-sul. Previous studies indicated that the catalytic mechanism of Egt2 involved covalent catalysis, where PLP needed to covalently bind to the protein before it could bind to the substrate. This suggested that Egt2 in *L. edodes* did not form a covalent bond with PLP. Interestingly, when we mutated the 247th amino acid residue of Egt2 from *L. edodes* to Lysine [12], based on the crystal structure of Egt2 from *Neurospora crassa*, we found that not only could the mutated Egt2 form a covalent bond with PLP, but the resultant Egt2-PLP complex was also capable of binding to the substrate Her-sul. 

**Figure 6:**
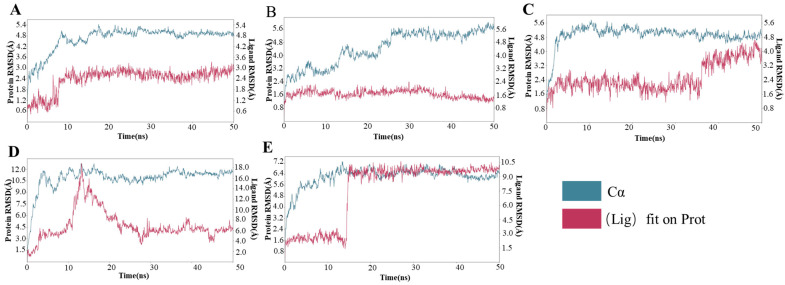
Molecular dynamics in the synthesis pathway of EGT in *L. edodes* bind to the amino acid sites and modes of action of substrates. (**A**): Egt1-Fe^2+^-His; (**B**): Egt1-Fe^2+^-SAM; (**C**): Egt1-Fe^2+^-Cys; (**D**): Egt2-PLP; (**E**): Egt2-PLP-Her-sul.

To delve into the stability of enzyme-substrate binding, we conducted 50 ns molecular dynamics simulations on the key enzyme Egt1 and its substrates His, SAM (S-adenosylmethionine), and Cys (cysteine) in the EGT synthesis pathway of *L. edodes.* The simulation results are presented in Figure 6. It was evident from the figure that after 10 ns, the root mean square deviation (RMSD) values of both Egt1 and His stabilized, indicating a robust binding between Egt1 and His. Upon binding with SAM, the RMSD value of SAM gradually stabilized, whereas the RMSD value of Egt1 exhibited an upward trend. This could be attributed to Egt1’s role as a methyltransferase, which transferred the methyl group from SAM to His during the reaction. Since the binding site of Egt1 on SAM was located near the methyl group, the loss of this methyl group weakened the steric hindrance at the Egt1-SAM binding region, leading to increased conformational fluctuations in Egt1. Contrastingly, in the Egt1-Cys system, the situation differed markedly from the Egt1-SAM system. During the molecular dynamic simulation, the RMSD value of Egt1 remained relatively stable, while the RMSD value of Cys suddenly rose in the later stages of the simulation and subsequently stabilized. This phenomenon might be due to the binding of Cys with Hercynine under the catalysis of Egt1, resulting in substantial conformational fluctuations.

Based on the results of molecular dynamics simulations, it was evident that the binding between the Egt2 and PLP exhibited a relatively stable state. Although some minor fluctuations in the RMSD values of PLP were observed during the simulation timeframe of 10 ns to 20 ns, these did not significantly impact the overall binding stability. However, when we introduced a point mutation in Egt2, specifically replacing the amino acid residue at position 247 with lysine (Lys), and subsequently conducted molecular dynamics simulations on the complex formed with PLP and Her-sul, we were surprised to find that during the same timeframe of 10 to 20 nanoseconds, the RMSD values of Her-sul showed a sharp increase, followed by a gradual stabilization towards a new steady state. The reasons behind this phenomenon might include the following points: (1) Her-sul had undergone significant conformational changes during this timeframe; (2) throughout the simulation process, Her-sul continued to adapt to its surrounding environment; (3) Her-sul had not yet reached a true stable state; (4) the intramolecular and intermolecular interactions reached a new equilibrium point. 

The aforementioned results indicated that Egt2, even after covalently binding with PLP, appeared unable to stably bind with Her-sul. The molecular docking result revealed that although Egt2 could bind with PLP, the binding mode was also non-covalent. This finding suggested that Egt2 and PLP in *L. edodes* may not bind covalently, implying that the catalytic mechanism of Egt2 in *L. edodes* may not be covalent catalysis.

## 3. Materials and Methods

### 3.1. Heterologous Expression Protein in Escherichia coli

pET-28 (+) vector was used for protein expression, transferring the constructed protein expression vector into the receptive state of *Escherichia coli* BL21 (DE3). The seed inoculum was inoculated in a liquid LB medium containing kanamycin, shaken at 37 °C for 200 r/min to activate to OD_600_ of 0.6–0.8, and IPTG was added to a final concentration of 0.5 mmol/L, and induced at 16 °C for 16 h. After collecting the bacterial cells, 4 mL of lysis solution (50 mM Tris HCl, 500 mM NaCl, pH 8.0) was added to resuspend on ice. The bacterial cells were crushed by ultrasound in an ice bath, the sonicated cells were taken and centrifuged at 4 °C, the clean supernatant collected, and the protein was purified using an imidazole solution.

### 3.2. Detection of EGT

The reaction pathway was reconstructed in vitro by referring to the synthesis pathway of EGT in fungi. The substrate solutions of Histidine (His), Cysteine (Cys), and S-Adenosyl Methionine (SAM) were prepared, and FeSO_4_-7H_2_O and PLP solutions were prepared as coenzymes. In addition, Tris-Hcl (pH 8.0) was also prepared as a buffer solution. The purified protein was added to the prepared reaction system, and after a period of reaction, the formation of EGT and intermediate products in the reaction products were detected using liquid chromatography-mass spectrometry (LC-MS). The conditions for liquid chromatography (LC) were as follows: the chromatographic column model was C_18_; at a temperature of 25 °C, the mobile phase A was a 0.1% formic acid acetonitrile solution, and the mobile phase B was a 0.1% formic acid aqueous solution. The mass spectrometry (Waters Vion IMS Qtof, Milford, MA, USA ) adopted a positive ion mode.

### 3.3. Molecular Docking

In this instance, the molecular docking process was executed using Schrödinger software (version 2021-4). The Maestro interface (version 13.0), which served as Schrödinger’s graphical user interface, was utilized for importing structures in an SDF format. LigPrep (version 3.4) was used to prepare ligands, involving the generation of 3D structures due to its great flexibility in use [24]. We carried out the following processing and settings based on the references, energy minimization with the OPLS2005 force field, protonation at pH 7.0 ± 2.0, and the creation of tautomers and stereoisomers. The protein’s 3D structure was imported into Maestro for preprocessing, which encompassed bond correction, residue completion, hydrogen addition, water molecule removal, and the elimination of other heteroatoms except for ligands. Energy minimization of the protein was then carried out using the OPLS2005 force field. The preprocessed and energy-minimized protein structure was utilized to generate a receptor grid (20 × 20 × 20 Å) for virtual screening based on molecular docking via the “Receptor Grid Generation” panel in the Glide module [25,26]. Maestro (version 13.0) was also leveraged to graphically depict interactions between compounds and pancreatic lipase.

### 3.4. Molecular Dynamics

To further analyze the binding mode of the active components with the target protein, molecular dynamics (MD) simulations were employed to investigate the interactions between enzymes and substrates. We conducted MD simulations utilizing Desmond (Version 6.0), following the methodology employed in our previous research [27], with the input file derived from the optimal conformation obtained through Glide molecular docking. Subsequently, the protein–ligand complex was solvated using the SPC water model [28], and ions (Na^+^ and Cl^−^) were added to maintain physiological conditions. In this study, an orthogonal box was utilized to impose periodic boundary conditions, and isothermal-isobaric (NPT) ensemble relaxation was applied to ensure system stability. The MD simulations were run for 50 ns, and the “Simulated Interaction Diagram” module was used for the analysis.

## 4. Conclusions 

In this study, we investigated the role of C-S lyase, a key enzyme involved in the formation of sulfur-containing odor components, in the synthesis of EGT in *L. edodes*. Through experimental and computational approaches, we found that the synthesis of EGT in *L. edodes*, similar to that in other fungi, was catalyzed by two key enzymes, Egt1 and Egt2, which acted on their respective substrates. *Lecsl*2, a C-S lyase, was involved in EGT synthesis in *L. edodes*. We also characterized the enzymatic catalytic mechanism of Egt1 and Egt2, the two enzymes primarily catalyzing EGT synthesis in fungi. The results showed that Egt1 interacted with His, SAM, and Cys to form the intermediate product Her-sul, while Egt2, a PLP-dependent enzyme, cleaved the C-S bond on Her-sul to produce EGT. However, we found that Egt2 in *L. edodes* may not form a covalent bond with PLP, unlike the previously reported catalytic mechanism of Egt2 involving covalent catalysis. 

Among the various edible fungi, including *L. edodes*, EGT is a common component [29,30]. Yet, it is *L. edodes* alone that possesses a unique sulfur-laden aroma. Prior research has widely held that the formation of this distinctive sulfur aroma in *L. edodes* was primarily attributed to the significant roles of two key enzymes, GGT and C-S lyase. Our investigation has uncovered that C-S lyase in *L. edodes* also acted as Egt2 in the synthesis of EGT. While the Egt2 gene is present in other edible fungi, they fail to produce the sulfur aroma distinctive to *L. edodes*. This indicates that Egt2 is not the pivotal factor accounting for the flavor disparity between *L. edodes* and other edible fungi. In addition to EGT, *L. edodes* contains a plethora of bioactive compounds. Our discovery that C-S lyase in *L. edodes* participated in EGT synthesis suggested potential avenues for utilizing *L. edodes* in future food processing to create products that are not only nutritious but also endowed with exceptional flavor.

In this study, we found that EGT synthesis in *L. edodes*, like other fungi, was catalyzed by Egt1 and Egt2, with Egt2 also crucial for forming unique sulfur-containing odor compounds. Unlike other fungi, *L. edodes* produces these distinct compounds. By selectively breeding or genetically modifying *L. edodes* to enhance Egt1 and Egt2 activity, we can increase the production of desirable sulfur-containing aroma compounds and EGT, enhancing their sensory quality and market value. Optimizing cultivation conditions through Egt1 and Egt2 expression can further boost production. Additionally, Egt2’s dual role suggests its potential biotechnological applications in ergothioneine and in sulfur-containing compound synthesis for pharmaceuticals, cosmetics, and food processing.

## Data Availability

The data presented in this study are available on request from the corresponding author.
